# Meta-analysis examining the association between low platelet monoamine oxidase levels and Zuckerman's sensation seeking scale in a sex dependent manner

**DOI:** 10.3389/fpsyg.2025.1544408

**Published:** 2025-02-10

**Authors:** Jordan Winfield, Ian A. Mendez, Gabriel A. Frietze

**Affiliations:** ^1^Pharmacy and Therapeutics, University of Pittsburgh School of Pharmacy, Pittsburgh, PA, United States; ^2^Department of Pharmaceutical Sciences, School of Pharmacy, The University of Texas at El Paso, El Paso, TX, United States

**Keywords:** monoamine oxidase (MAO), sensation seeking, impulsive, Zuckerman's sensation seeking scales, neurobiology, meta-analysis

## Abstract

Monoamine oxidase (MAO) is an enzyme in the brain responsible for breaking down neurotransmitters. MAO levels can be measured in humans by collecting blood platelets. Low platelet MAO levels in healthy individuals are associated with personality differences, such as increases in sensation seeking. In this meta-analysis, we investigated the association between low platelet MAO activity and sensation seeking behavior, as measured by Zuckerman's Sensation Seeking Scale (SSS). To identify studies to include in this meta-analysis, a preliminary database was produced by searching PsycInfo, Medline, PubMed, ProQuest, and ScienceDirect, from the time period of January 01, 1970 through August 01, 2022. The Preferred Reporting Items for Systematic Reviews and Meta-Analyses (PRISMA) guidelines were followed for study inclusion. Fourteen studies with a total of 24 correlations and 1,470 participants were included in the analyses. Across 24 independent effect sizes, the correlations ranged from −0.74 to 0.40. The Random Effects Model (REM) yielded a weighted average correlation of −0.22 (95% CI = −0.31, −0.13), indicating an inverse relationship where lower levels of MAO tend to be associated with higher levels of SSS. A subgroup analysis was used to examine the effects of gender. The REM yielded a weighted average correlation of −0.22 (95% CI = −0.33, −0.10) for the effect sizes of males, −0.22 (95% CI = −0.47, 0.06) for the effect sizes of females, and −0.23 (95% CI = −0.38, −0.06) for the effect sizes that included both males and females. The subgroup analysis did not reveal differences between males and females on the association between human blood platelet MAO levels and SSS. Our hypothesis that there is a negative association between MAO levels and SSS was supported. These findings have potential clinical implications suggesting that MAO platelet concentrations could be used as a potential biomarker for identifying maladaptive behaviors.

## Introduction

Monoamine oxidase (MAO) is an enzyme in the brain that is found on the outer membrane of mitochondria found in cell bodies. This includes neuronal cell bodies, where it is responsible for regulating monoamine neurotransmitters (Zuckerman, 1994b; Shih et al., 1999). Specifically, MAO breaks down neurotransmitters, like serotonin and dopamine, in both the presynaptic neuron and within the synaptic cleft. MAO levels have been measured in humans by collecting blood platelets (Zuckerman, 1994b; Zuckerman and Kuhlman, 2000) and appear to be genetically determined (Nies et al., 1973) and stable over time (Murphy et al., 1976). Importantly, MAO levels have been found to be negatively associated with a number of disorders, including drug dependence, attention deficit disorder, bipolar disorder, schizophrenia, and suicidal behavior (Fowler et al., 1982; Buchsbaum et al., 1977; Murphy et al., 1982). Notably, many of the disorders that are associated with MAO are characterized by an inability to control impulses or anticipate negative consequences of behavior, also referred to as disinhibitory psychopathology (Reist et al., 1990).

Low platelet MAO levels in healthy individuals are associated with personality differences, such as increases in sensation seeking (Schooler et al., 1978; Zuckerman, 1994a). Sensation seeking is defined as the extent to which people seek experiences that are stimulating and novel (Zuckerman, 1994a, p. 27). One of the most common measures for assessing sensation seeking is the *Zuckerman Sensation Seeking Scale* (SSS; Zuckerman, 1971). The SSS contains four components that together make up a total sensation seeking score, labeled as General SSS. The first component is *Thrill and Adventure Seeking* and measures the extent to which people seek exciting or thrilling experiences, such as skydiving (Zuckerman, 1971). The second component is *Experience Seeking* and measures the extent to which people seek novel experiences, such as traveling somewhere they have never been (Zuckerman, 1971). The third component is *Disinhibition* and measures the extent to which people seek situations that are disorderly or unmanageable, such as running with the bulls (Zuckerman, 1971). The fourth and final component is *Boredom Susceptibility* and measures the extent to which people can withstand boring situations, such as waiting in a doctor's office (Zuckerman, 1971).

Research using the SSS has demonstrated with varying degrees that sensation seeking was attributed to lower MAO activity, inferred from observed decreases in MAO activity with isotopic methods measuring MAO activity *in vitro*. Key studies have yielded major findings that highlight a potential biological predisposition to engage in sensation seeking and risky behaviors. In a study by Fowler et al. (1980), individuals who were interested in mountaineering were identified as sensation seekers (as indexed by the Zuckerman SSS) and had lower platelet MAO levels (*p* < 0.05), indicating a negative association between MAO levels and sensation seeking. Similarly, in a study by Carrasco et al. (1999) investigating bullfighters, the authors found that bullfighters were identified as sensation seekers and had lower platelet MAO levels (*p* < 0.05). The authors concluded that there might be a correlation between a tendency to partake in risky behaviors and sensation seeking, with the potential involvement of biological elements of personality that are evidenced by reduced platelet MAO levels (Carrasco et al., 1999). Another study sought to examine biochemical and personality correlates in college students to understand how the frequency of alcohol consumption is impacted (La Grange et al., 1995). The authors found that low MAO levels and high sensation seeking contributed to variability in alcohol consumption in males, however, MAO levels did not contribute to variability in alcohol consumption in females. Importantly, Zuckerman (1994a) compiled research conducted from 1977 to 1990, examining the negative correlation between low human blood platelet counts of MAO and increased sensation seeking as determined by the General SSS, and noted that 9 out of 13 correlations were statistically significant. Interestingly, negative correlations are more consistently observed in males than females (Calhoon-La Grange et al., 1993).

While finding have been mixed, some studies suggests that weak or inconsistent correlations between MAO platelet levels and sensation seeking in females could be driven by hormonal fluctuations (Calhoon-La Grange et al., 1993). Specifically, endocrine factors are known to affect MAO platelet levels, thus, hormonal influences could be attributable as a factor that mediates the association between MAO and sensation seeking. Furthermore, the gene that codes the production of MAO is found on the X-chromosome, thus, females naturally produce more MAO than males (Robinson et al., 1971; Shih et al., 1999). Further research is warranted to examine sex differences in MAO and SSS to make sense of the inconsistent findings in males and females.

In this meta-analysis, we investigated the association between low platelet monoamine oxidase (MAO) activity and sensation seeking behavior, as measured by Zuckerman's Sensation Seeking Scale (SSS), with a focus on gender differences. The present meta-analysis will yield a better understanding of the neurobiological basis of sensation seeking and has implications for understanding if a blood platelet biomarker test could have potential for predicting personality traits. Furthermore, this meta-analysis may help clinicians comprehend and potentially treat maladaptive sensation-seeking behaviors. We analyzed studies that compared individuals with low vs. normal/high MAO activity and their corresponding SSS scores. Additionally, we explored the impact of this association on gender by comparing findings between males and females. The hypothesis of the current meta-analysis is that there is a negative association between MAO levels and SSS. The research questions we addressed were: (1) what is the association between low platelet monoamine oxidase levels and sensation seeking behaviors as indexed by Zuckerman's sensation seeking scale, and (2) does gender have an impact on the association between monoamine oxidase and sensation seeking behavior?

## Materials and methods

To identify studies to include in the meta-analysis, a preliminary database was produced by searching PsycInfo, Medline, PubMed, ProQuest, and ScienceDirect, from the time period of January 01, 1970 through August 01, 2022. Snowballing (i.e., pursuing references from acquired articles' references). A search of unpublished theses and dissertations was also conducted.

The Preferred Reporting Items for Systematic Reviews and Meta-Analyses (PRISMA) guidelines were followed for study inclusion (Moher et al., 2009). Combinations of the following key terms were searched in the abstract, title, and throughout the entire text: *monoamine oxidase, MAO, biological correlate, biochemical correlate, and enzyme* combined with the terms *sensation seeking, SSS, Zuckerman, impulsive, inhibition, risk behavior, and personality*. Boolean search operators were included for “sensation seeking” and “Zuckerman”. The earliest study found was from 1977 and the latest study was in 2022. No funding was provided to carry out this meta-analysis.

### Specific search engine results

**Figure 1:**
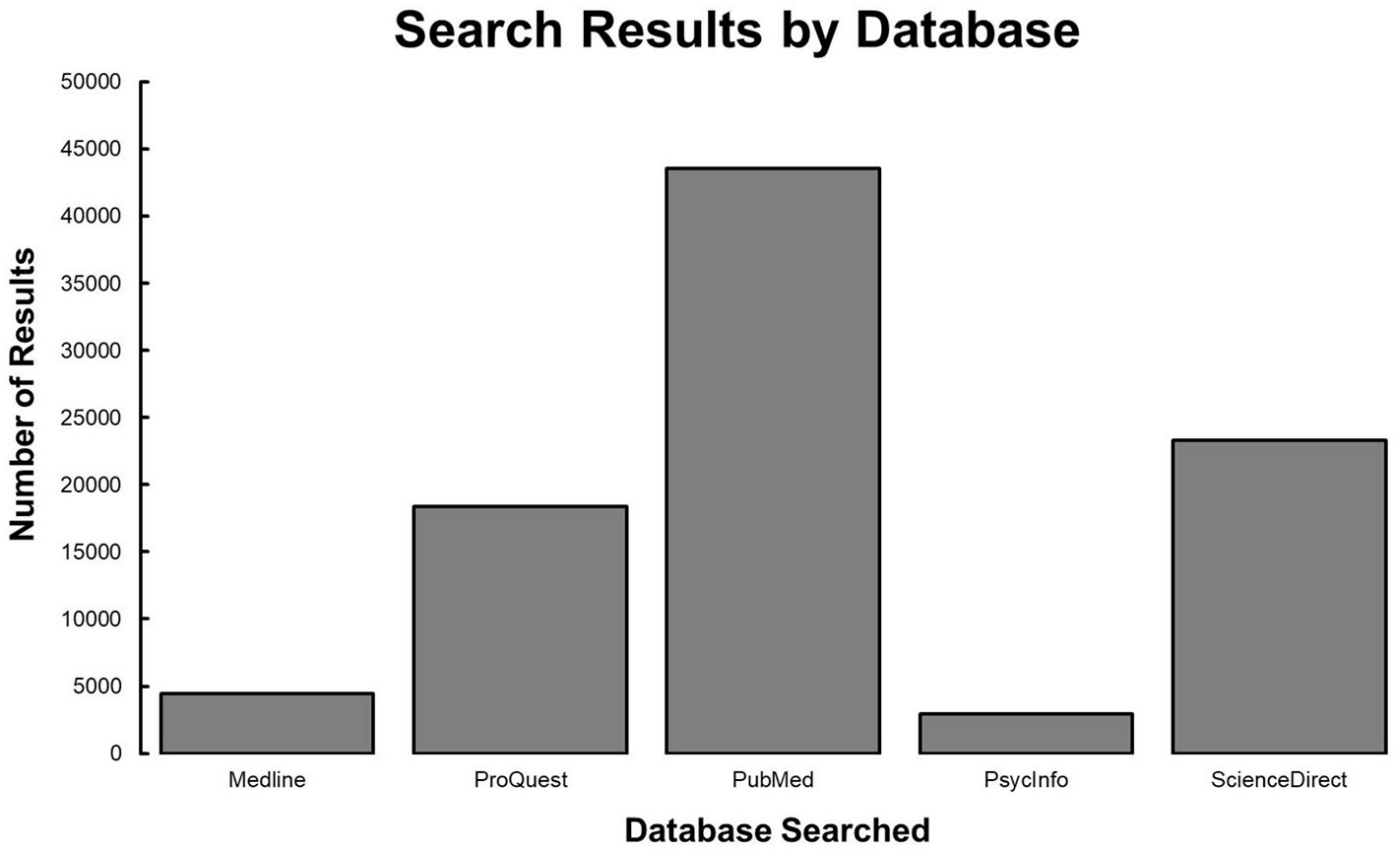
Frequency of MAO and SSS search results.

PubMed, PsycInfo, Medline, ProQuest, and ScienceDirect were the selected databases used for this study. PsycInfo and Medline were searched in EBSCO by selecting the option to search the specific database. Each database was searched separately in EBSCO to keep track of the number of records obtained for each database. Regarding PsycInfo and Medline these search procedures yielded 2,938 and 4,469, respectively. These references were extracted as a Research Information Systems (RIS) Formatted File and uploaded to EndNote 20. ProQuest, a platform for searching officially completed academic Thesis and Dissertations, was searched to prevent publication bias. The same key terms stated above were searched in combination using Boolean search operators in the full text and yielded 18,374 studies. These references were extracted as a RIS Formatted File and uploaded to EndNote 20. ScienceDirect and PubMed were also searched using Boolean search operators with our key terms. Total articles when searching the entire text yielded 23,328 and 42,575 results in ScienceDirect and PubMed, respectively (see Figure 1). These references were extracted as a RIS Formatted File and uploaded to EndNote 20.

### Exclusion criteria

**Figure 2:**
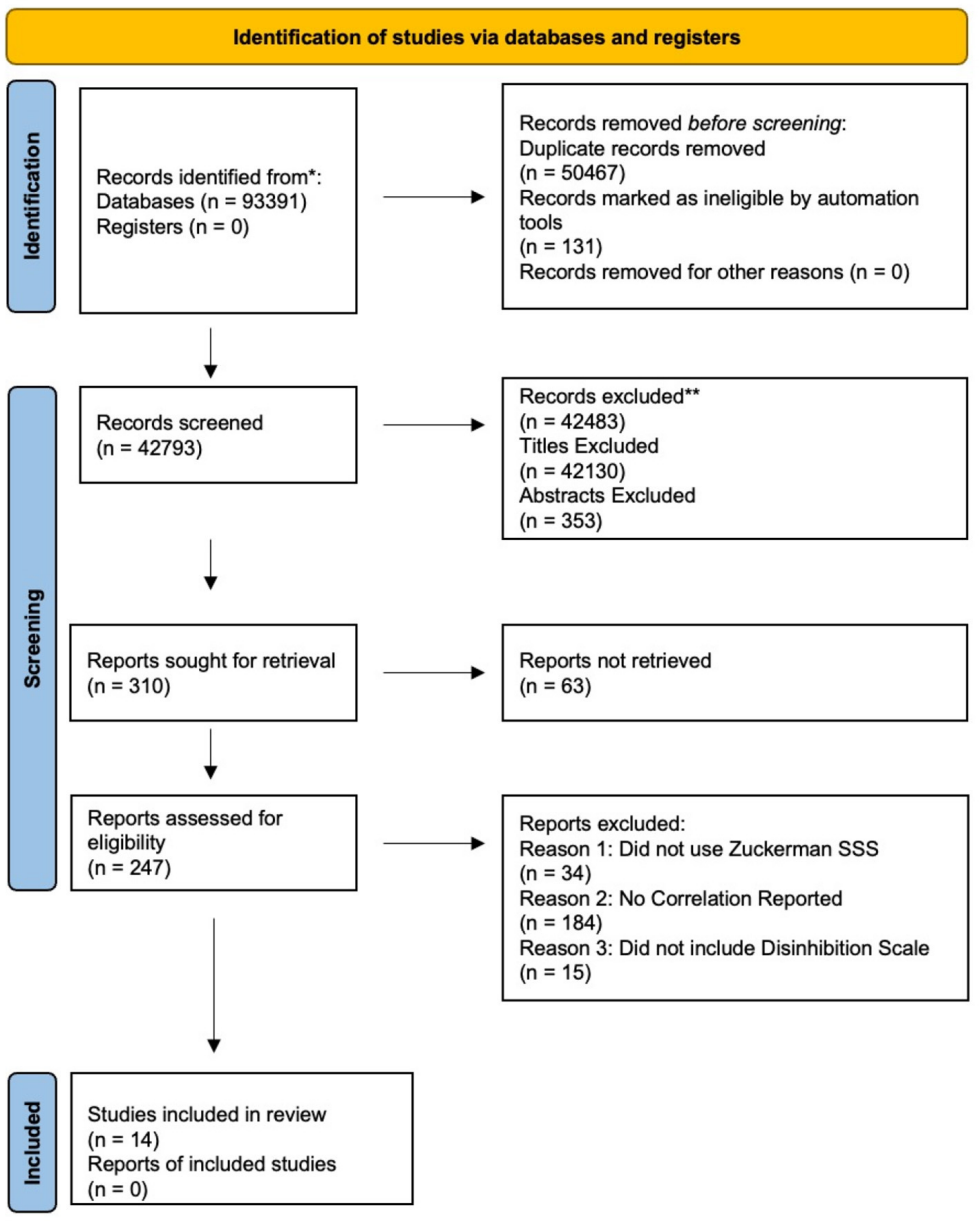
PRISMA flow diagram. ^*^The number of records identified from each database or register searched is provided in Figure 1. ^**^No records were excluded by automation tools.

Studies were excluded for three reasons: (1) did not use Zuckerman's SSS for measuring sensation seeking, (2) used the shortened version of the Zuckerman scale that does not contain the disinhibition subscale, which is reported as one of the strongest correlates of MAO (Zuckerman, 1994a, p. 298), or (3) did not publish the correlation in the paper or enough information necessary to calculate the correlation. The citation and reference management software EndNote 20 was used to organize and manage all references obtained from the literature search. Duplicate records and retractions were removed within EndNote to ensure data accuracy. After removing duplicates and retractions we established a PRISMA guideline workflow within EndNote 20 to manage and sort all studies (Figure 2). First, we performed a title and abstract exclusion approach to eliminate studies that were focused on disease states that are irrelevant and those focusing on animal studies, cell and tissue studies, and compound synthesis not related to behavior in human subjects. We kept studies focusing on behaviors relating to reward or sensation seeking; excluding irrelevant results and keeping potential articles for further screening that consisted of sensation seeking, reward, sex, alcohol, gambling, addiction, impulsivity, dopamine, and behavior. We performed the same exclusion approach for studies that did not focus on illicit drug use and addiction, hedonistic behaviors, and self-indulgent behaviors such as overeating, gambling, and sex. Lastly, we screened all titles and excluded those not focused on correlation and association concerning monoamine oxidase and behaviors including gene expression. The same systematic method of exclusion was performed to exclude the abstracts.

### Inclusion for meta-analysis data

Approximately 310 records were sought for retrieval. Of these 310 records, 63 abstracts were linked to inaccessible PDFs or online links to view the full text. To obtain the remaining studies lacking the PDFs a search was completed in Google Scholar. If the articles could not be retrieved through Google Scholar but had enough information in the abstract to support paying for the article, requests were made through our university librarian to purchase the article. The remaining, 247 studies were initially obtained because they measured platelet MAO and/or sensation seeking. Of these studies, 34 did not use the Zuckerman SSS to measure sensation seeking but rather an alternative psychometric questionnaire developed to measure either sensation seeking or impulsivity which is highly correlated with sensation seeking. Another 15 studies did not include a disinhibition subscale in the Zuckerman SSS. The remaining 184 studies met the initial inclusion criteria of testing MAO and SSS, however, they failed to include the correlation between MAO and SSS or the necessary information to calculate an estimated correlation.

Overall, seven studies included in the present meta-analysis were obtained from searching PsycInfo and Medline simultaneously through EBSCO. Moreover, three studies were obtained from Science Direct and three studies from PubMed. Only one study was obtained from ProQuest. A total of 24 correlations were retrieved from the above-mentioned studies and synthesized in the present meta-analysis. To ensure that high quality studies were evaluated in the present meta-analysis, all of the studies were thoroughly reviewed by the research team to ensure that they met the inclusion criteria.

### Approach to analysis

Computations in the current study were carried out using Comprehensive Meta-Analysis Version 4 (Borenstein et al., 2022). Correlations were extracted from studies examining the association between blood platelet MAO and the Zuckerman SSS. A Fixed Effects Model (FEM) and a Random Effects Model (REM) were used to calculate the weighted average correlation in the studies. The FEM assumes that the studies included in the analysis are drawn from the same underlying population, employ identical methodologies, and are estimating the same treatment effect (Borenstein, 2019; Borenstein et al., 2010, 2021; Hedges and Olkin, 1985; Hedges and Vevea, 1998; Higgins and Thomas, 2019). Alternatively, the REM model assumes that there are differences between the population, methodologies, and are estimating varying treatment effects across studies (Borenstein, 2019; Borenstein et al., 2010, 2021). As part of the FEM, the Q-test was calculated to examine if there was evidence of any variations in effect size across studies. I-squared was calculated to understand the proportion of the variance within the observed effects that reflects variation in the true effects rather than sampling error. Tau-squared was calculated to examine the variance of the true effects and the prediction interval was calculated to provide a range of within which the results of a future study might fall if the study was selected at random from the same underlying population. A variety of methods were also used to assess publication bias, including inspecting a funnel plot, calculating the classic fail-safe *N*, Begg and Mazumdar Rank Correlation Test, Egger's Test of the Intercept, and Duval and Tweedie's Trim and Fill.

### Transparency and openness

The data for this meta-analysis will be made available by contacting the corresponding author. The study design and analytical plan were not preregistered.

## Results

### General SSS

#### Fixed effects model (REM) and random effects model (REM)

**Figure 3:**
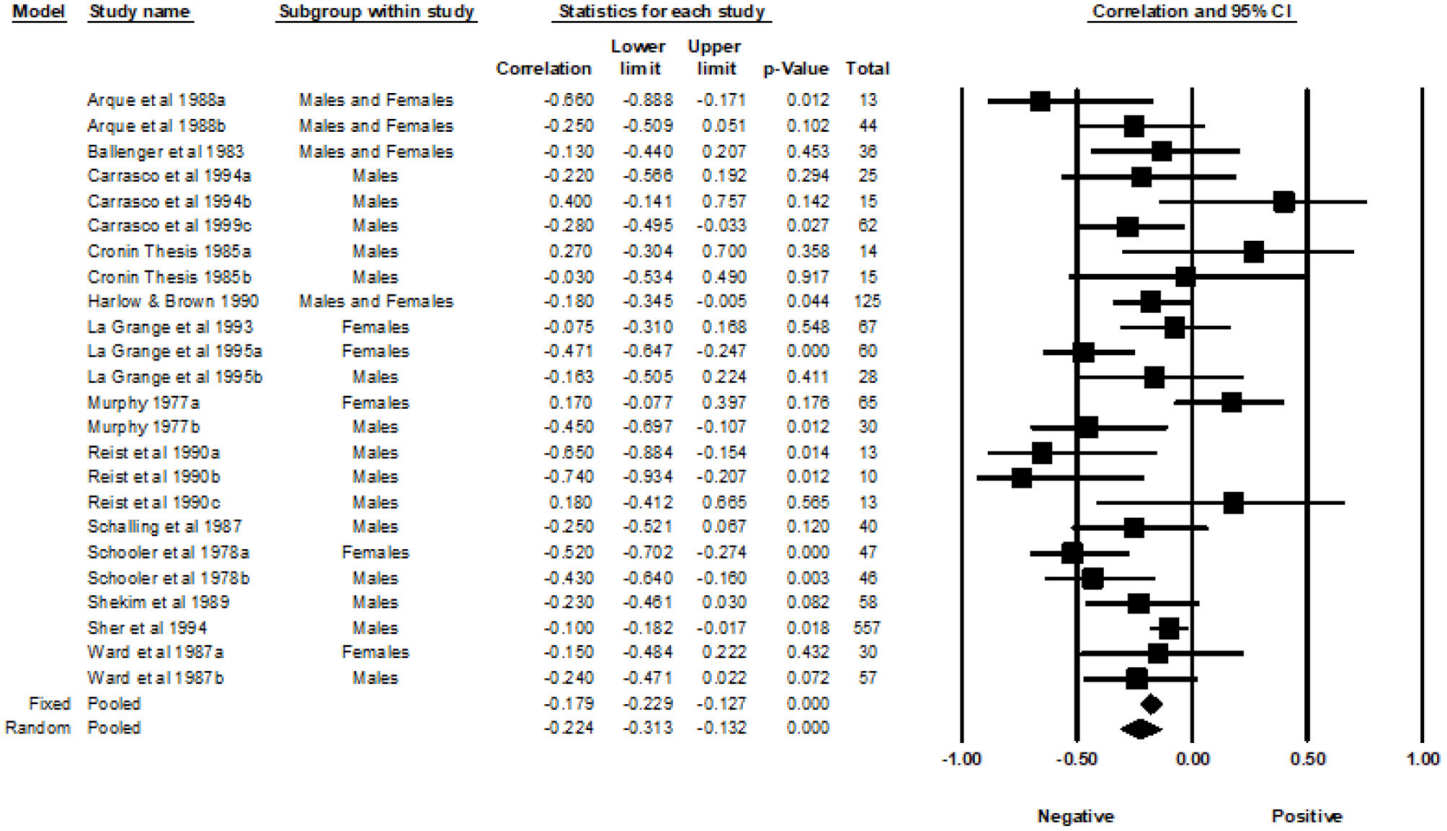
Forest plot of correlations between general SSS and MAO.

Fourteen studies from 1977 to 1999, with a total of 24 correlations and 1,470 participants, were included in the analyses examining the association between the General SSS and MAO. Across the 24 independent effect sizes, the correlations ranged from −0.74 to 0.40. Figure 3 provides a forest plot summarizing the correlations observed and provides a visual comparison of the studies analyzed. The Fixed Effects Model (FEM) yielded a weighted average correlation of −0.18 (95% Confidence Interval [CI] = −0.23, −0.13). The heterogeneity test indicated that one or more of the 24 effect sizes may not represent the same underlying population (*Q* = 52.30, *p* < 0.001, *I^2^* = 56.03). The I-squared value indicated that 56.03% of the variance in the observed effects reflect variance of the true effects rather than sampling error. The REM yielded a weighted average correlation of −0.22 (95% CI = −0.31, −0.13). The tau-squared was equal to 0.026 in Fisher's Z units and tau was equal to 0.160 in Fisher's Z units.

#### Prediction interval for general SSS and MAO

**Figure 4:**
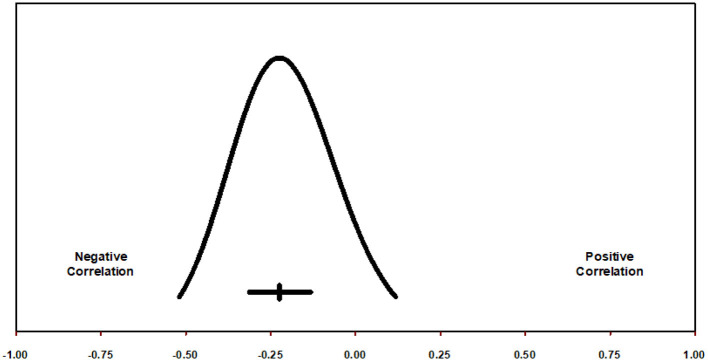
Prediction interval for correlation between general SSS and MAO.

If we assume that the true effects are normally distributed (in Fisher's Z units), we can estimate that the prediction interval is −0.519 to 0.118 (Figure 4) and that the true effect size in 95% of all comparable populations falls in this interval (Borenstein, 2019, 2020; Borenstein et al., 2021, 2017; Higgins and Thomas, 2019).

#### Publication bias

**Figure 5:**
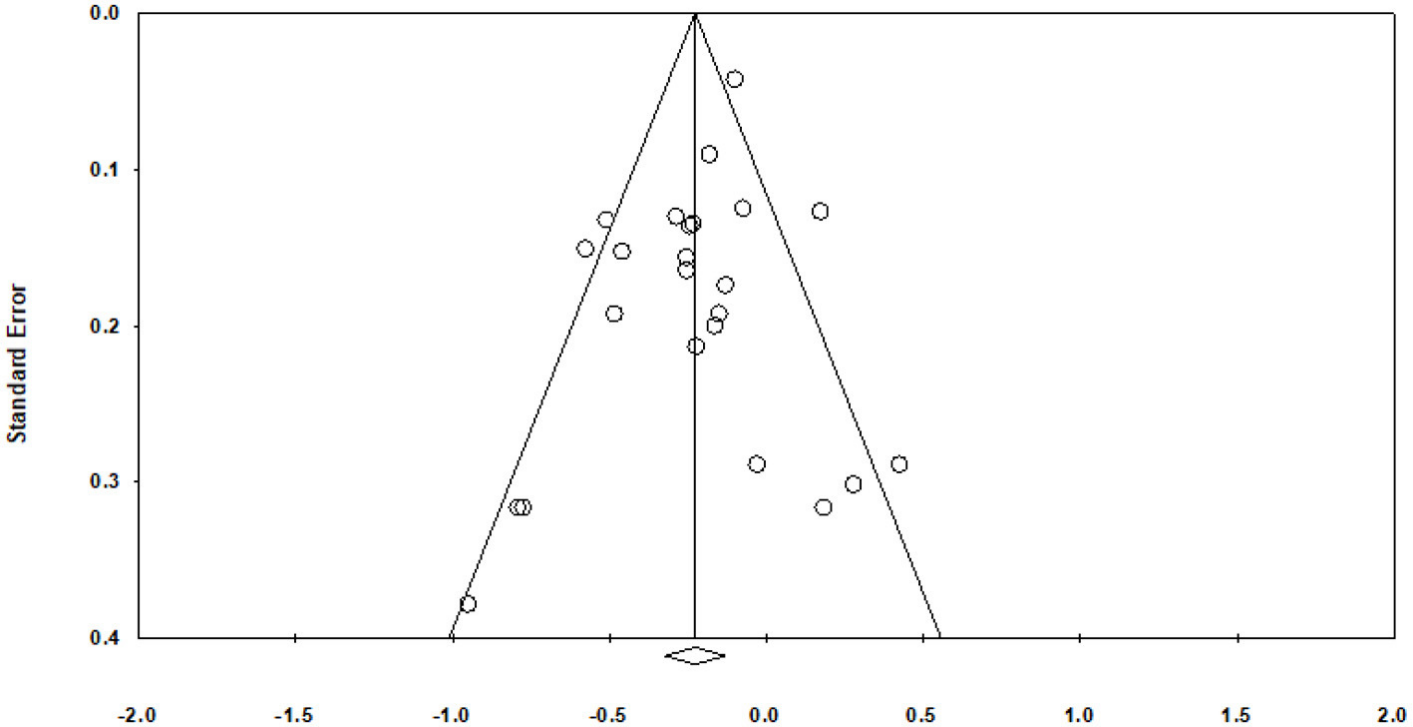
Funnel plot of standard error by Fisher's Z.

A funnel plot did not reveal concerns of publication bias (Figure 5). The results of the Classic Fail-Safe *N* test estimated that 274 missing studies with an effect size equal to would be needed to increase the *p*-value to above 0.05. The Begg and Mazumdar rank order correlation between the treatment effect and the standard error revealed that Kendall's tau b is −0.058 (one-tailed *p*-value = 0.34573; two-tailed *p*-value = 0.69146 based on continuity-corrected normal approximation). Egger's test of the intercept revealed that (*B*_0_) is −0.85083, 95% confidence interval (−2.00284, 0.30118), with *t* = 1.53, *df* = 22 (one-tailed *p*-value = 0.06993; two-tailed *p*-value = 0.13986). Duval and Tweedie's Trim and Fill method revealed that the fixed effect model point estimate and 95% confidence interval for the combined studies is −0.18 (−0.23, −0.13), with no studies being trimmed. The random effects model point estimate and 95% confidence interval for the combined studies was −0.22 (−0.31, −0.13), with no studies being trimmed.

#### Gender subgroup analysis

A subgroup analysis was used to examine the effects of gender. The male group included a sample of 15 effect sizes, the female group included a sample of five effect sizes, and the studies that included both males and females had a total of four effect sizes. The FEM yielded weighted average correlation of −0.16 (95% CI = −0.23, −0.10) for the effect sizes of males, −0.21 (95% CI = −0.32, −0.09) for the effects sizes of females, and −0.22 (95% CI = −0.34, −0.08) for the effect sizes of studies that included both males and females. Heterogeneity was detected in the male subgroup (Q= 26.46, *p* = 0.02, *I^2^* = 47.09) and the female subgroup (Q = 21.37, *p* < 0.001, *I^2^* = 81.28). Heterogeneity was not detected in the subgroup that included both genders (Q = 3.77, *p* = 0.287, *I^2^* = 20.45). The REM yielded a weighted average correlation of −0.22 (95% CI = −0.33, −0.10) for the effect sizes of males, −0.22 (95% CI = −0.47, 0.06) for the effect sizes of females, and −0.23 (95% CI = −0.38, −0.06) for the effect sizes that included both males and females.

### Thrill and adventure seeking

**Figure 6:**
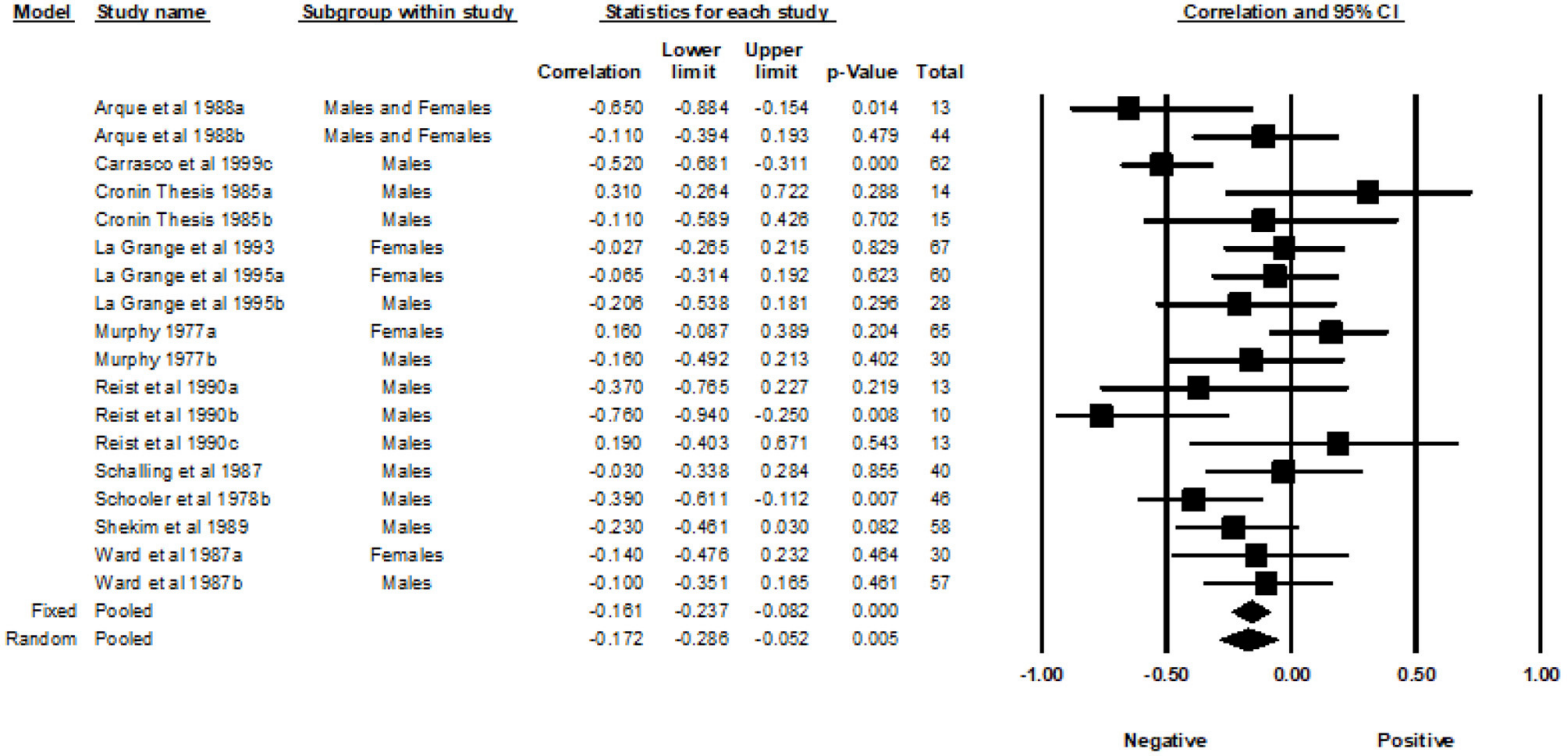
Forest plot of correlations between TAS and MAO.

A total of 18 correlations examined the association between the Thrill and Adventure Seeking (TAS) subscale and MAO (Figure 6). The REM yielded a weighted average correlation of −0.17 (95% CI = −0.29, −0.05).

### Experience seeking

**Figure 7:**
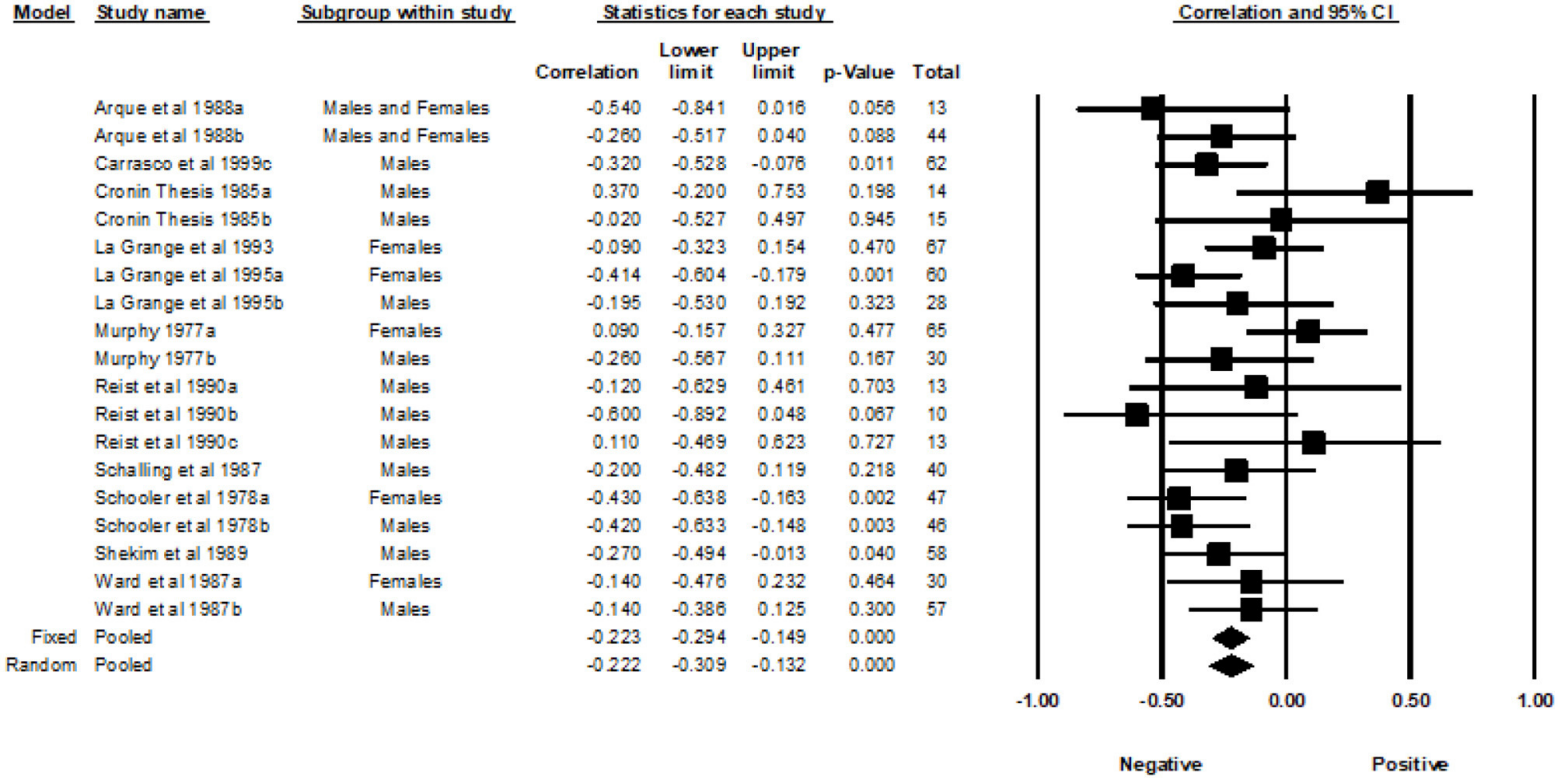
Forest plot of correlations between experience seeking and MAO.

A total of 19 correlations examined the association between the Experience Seeking subscale and MAO (Figure 7). The REM yielded a weighted average correlation of −0.22 (95% CI = −0.31, −0.13).

### Disinhibition

**Figure 8:**
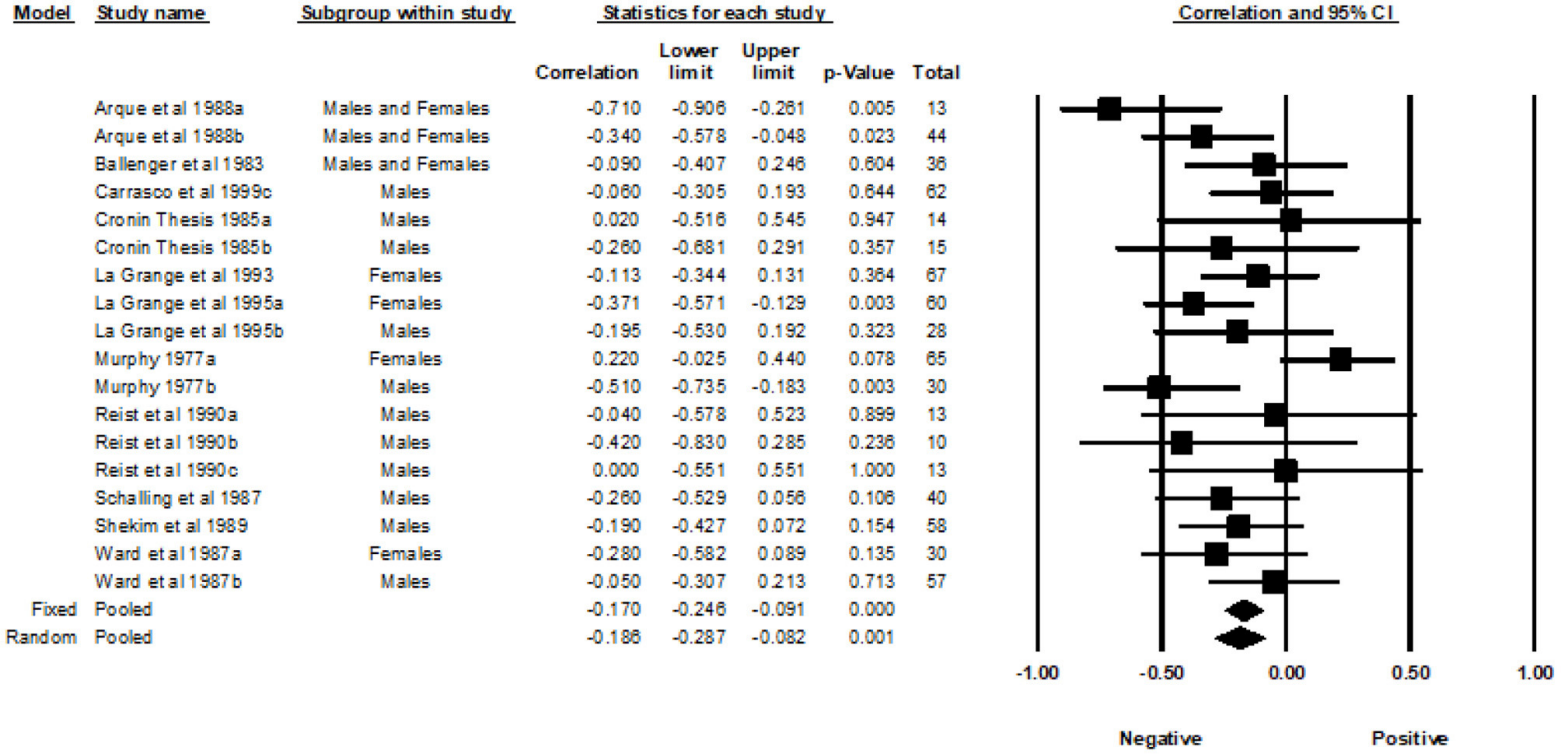
Forest plot of correlations between disinhibition and MAO.

A total of 18 correlations examined the association between the Disinhibition subscale and MAO (Figure 8). The REM yielded a weighted average correlation of −0.19 (95% CI = −0.29, −0.08).

### Boredom susceptibility

**Figure 9:**
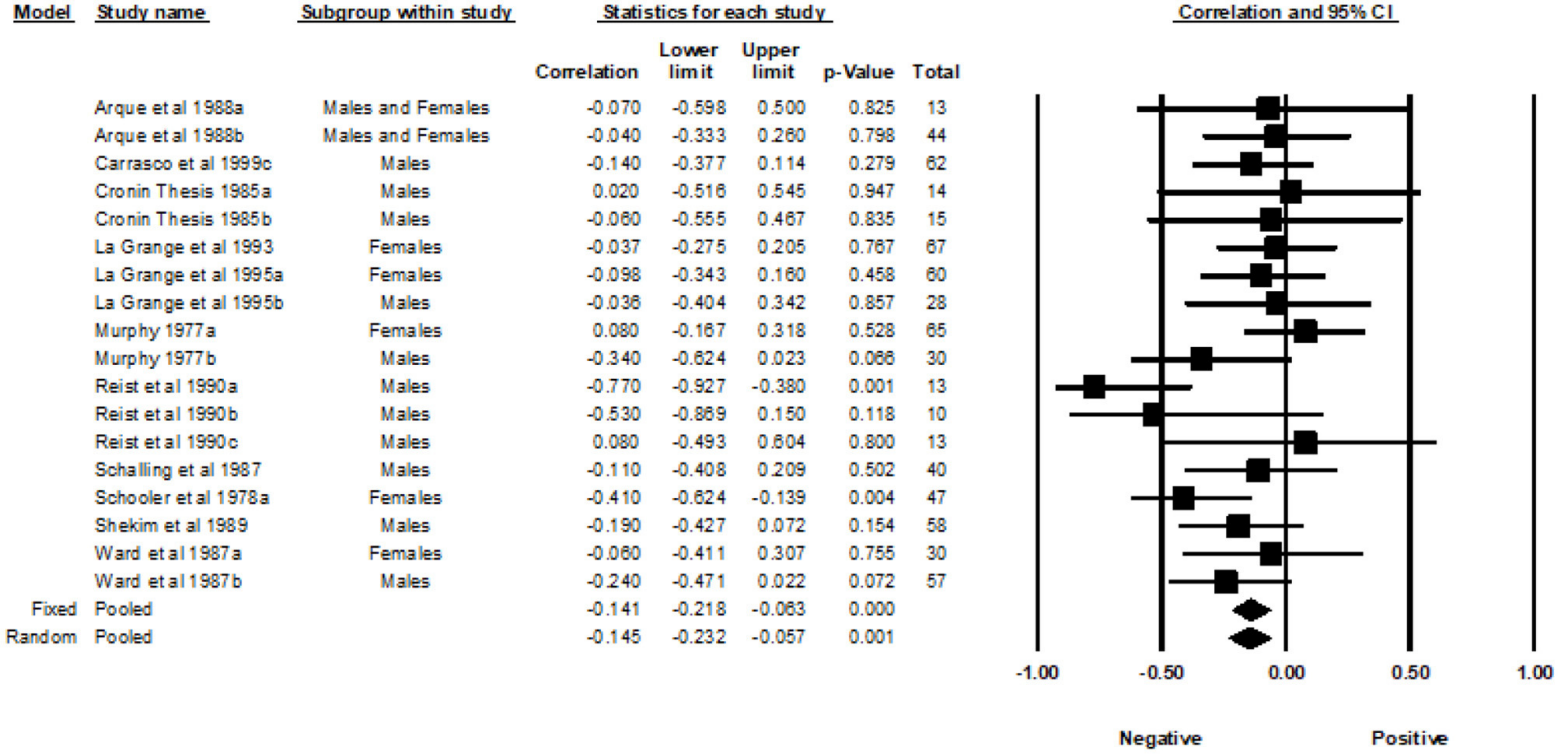
Forest plot of correlations between boredom susceptibility and MAO.

A total of 18 correlations examined the association between the Boredom Susceptibility subscale and MAO (Figure 9). The REM yielded a weighted average correlation of −0.15 (95% CI = −0.23, −0.06).

## Discussion

The primary goal of this meta-analysis was to evaluate the impact of MAO on SSS. Over three decades of research suggests that a negative association exists between human monoamine oxidase (MAO) blood platelet levels and sensation seeking. Our hypothesis that there is a negative association between MAO levels and SSS was supported. The negative correlation indicates that lower levels of MAO are associated with higher levels of sensation seeking. Conversely, high levels of MAO are associated with lower levels of sensation seeking. These findings are consistent with prior research examining the relationship between MAO and SSS (Zuckerman, 1994a). Moreover, these findings have potential clinical implications for treating individuals diagnosed with disorders related to impulsivity or sensation seeking including drug dependence, attention deficit disorder, bipolar disorder, schizophrenia, and suicidal behavior (Fowler et al., 1982; Buchsbaum et al., 1977; Murphy et al., 1982). Pharmacological interventions such as the use of MAO inhibitors might be a useful treatment to reduce sensation seeking and impulsivity in individuals diagnosed with these disorders.

Another key finding that emerged in the current meta-analysis is that our subgroup analysis did not reveal differences between males and females on the association between human blood platelet MAO levels and SSS. Prior research indicates that significant negative correlations are more consistently observed in males than in females (Calhoon-La Grange et al., 1993). Our findings suggest that there was a weak negative correlation in both the male and female subgroups. Our analysis indicated the female subgroup exhibited a slightly stronger negative correlation than the male subgroup. However, it is imperative to consider the presence of heterogeneity within the male and female subgroups, as highlighted by significant Q statistics and I^2^ values. Although the female subgroup demonstrated a slightly stronger negative correlation than the male subgroup it exhibited a higher degree of heterogeneity. This heterogeneity signifies variability in the results of studies within each subgroup. Regarding the mixed gender subgroup analysis there was a slight negative correlation between platelet MAO and SSS just as with the male and female subgroup. Interestingly, the absence of heterogeneity in the mixed-gender subgroup suggests that aggregating data from both genders yields a more consistent estimate of the effect size. Taking a closer look at the correlation differences between males and females it is important to note that previous literature has compared hormonal differences between high and low sensation seekers with regards to gender. Factor analysis of the relationships between sensation seeking traits and hormonal levels in males and females concluded that high sensation seekers exhibited significantly higher levels of testosterone, 17-B estradiol, and estrone compared to low sensation seekers (Daitzman and Zuckerman, 1980).

Furthermore, our study revealed significant associations between all four subscales of the SSS and MAO. Few researchers have reported a correlation between MAO and the Boredom Susceptibility subscale of the SSS (Shekim et al., 1989; Reist et al., 1990). In the current meta-analysis, the Boredom Susceptibility scale had the lowest weighted average correlation of the 3 subscales. In contrast, many researchers have found a correlation between MAO and the Disinhibition subscale of the SSS (Arqué et al., 1988; Murphy, 1977; Schalling et al., 1987; Shekim et al., 1989; Ward et al., 1987; Sher et al., 1994). We found that the strongest weighted average correlation was between MAO and the Experience Seeking scale.

While this meta-analysis provides evidence for understanding the neurobiological basis of sensation seeking, there are some limitations that should be addressed. One methodological constraint of the present meta-analysis was the extensive time required to conduct a thorough review of the literature due to the broad temporal range which extended as far back as the 1970s. This lengthy timeframe yielded a large number of search results, requiring significant effort to carefully screen and evaluate each paper to ensure they met the inclusion criteria. Future researcher might benefit from incorporating new technology that utilizes machine learning or artificial intelligence to increase the efficiency of screening a large number of papers for exclusion. The studies used in this meta-analysis do not differentiate between MAO A and B platelet activity with regards to their correlation to Zuckerman's SSS. Briefly, both enzymes are bound to the outer mitochondrial membrane and have 70% amino acid sequence identity (Gaweska and Fitzpatrick, 2011). It is important note that while MAO A primarily metabolizes serotonin, norepinephrine, and dopamine. MAO B preferentially oxidizes benzylamine and phenylethylamine (Gaweska and Fitzpatrick, 2011). However, detailed analysis of both enzymes' crystal structure at high resolution demonstrates that despite differences in binding specificity MAO B does metabolizes dopamine, norepinephrine, serotonin just not as efficiently as MAO A (Son et al., 2008). As such, the correlation seen with platelet activity and sensation seeking could be a result of MAO A or MAO B separately or a combination of both enzymes.

The studies used in this meta-analysis focus primarily on platelet MAO levels and correlate the levels with Zuckerman SSS. However, it could be argued that a more appropriate bioanalytical parameter would be MAO activity in the brain. Literature supports the use positron emission tomography (PET) imaging, to examine the distribution and levels of MAO in the human brain (Tong et al., 2013). The outcomes of PET imaging using specific radiotracers are commonly correlated with the protein levels in postmortem human brains (Tong et al., 2013). It is significant to note that while this supports PET imaging as a clinical and research tool, there are some shortcomings. Firstly, this technique only correlates the MAO levels that can be quantified from postmortem brain tissue which does not give us much information about activity. Furthermore, this methodology underestimated the regional contrast of MAO distribution by two-fold compared to *in vitro* data (Tong et al., 2013). A more reliable and efficient method for analyzing MAO activity in clinical samples, supporting both research and therapeutic monitoring applications, is high-performance liquid chromatography (HPLC). Without the use of radiotracers, HPLC only uses 2 mls of plasma to obtain data of platelet MAO activity (Ogata et al., 1992). This technique has been proven to be sensitive and suitable for clinical trials than other methods. HPLC as a bioanalytical tool gives true activity of the MAO. Regarding MAO expression, localization, and activity levels as it pertains to quantification for *in vivo* and clinical studies, it is important to note that the distribution and relative activity of MAO A and MAO B vary between different tissues. Literature suggests that despite similar quantities of MAO A and MAO B proteins in many tissues, MAO A tends to be the dominant form in terms of activity (Billett, 2004). This is attributed to its higher molecular turnover rate with most substrates, except phenylethylamine and benzylamine (Gaweska and Fitzpatrick, 2011; Billett, 2004). Although, some studies used in this meta-analysis do not differentiate between MAO A and MAO B, platelets predominately contain MAO B. mRNA and protein expression levels are independent of each other and of protein activity due to post transcriptional and post translational modifications to mRNA and protein, respectively (Grimsby et al., 1990). There has been a long-held assumption that platelet MAO activity reflects the same activity in the brain, which has been a basis for using platelet MAO as a biomarker in neuropsychiatric research (Tong et al., 2013). This approach is evident in the studies used in this meta-analysis. However, a clinical study of patients with epilepsy undergoing neurosurgery illustrated that while there is a significant positive correlation between cerebral cortical MAO A and MAO B activity there is no significant correlation between the activity of MAO B in the cerebral cortex and platelets (Young et al., 1986). Analogously, platelet MAO B activities did not significantly correlate with cerebral cortical MAO A activities. Although the lack of correlation found in this study suggests caution in interpreting or relying on platelet MAO measurements as indicative of brain MAO activity or psychopathological conditions, this patient population had been treated with medications for epilepsy, which could influence MAO activity (Young et al., 1986). Furthermore, the data presented was from a small sample size. Lastly, it is important to note that confounding variables such as age and sex may also influence MAO activity.

Two major dimensions have been identified by factor analysis; Stable Extraversion vs. Neurotic Introversion and Social Deviancy vs. Social Conformity. Testosterone and Estradiol both demonstrated distinct patterns of correlation with personality traits, aligning with the factor analysis dimensions (Daitzman and Zuckerman, 1980). These results concluded that testosterone is correlated positively with sociability, extraversion, and heterosexual interest/experience, while negatively correlating with neuroticism, introversion, femininity, socialization, and self-control (Daitzman and Zuckerman, 1980). Estradiol correlated positively with psychotic and psychopathic traits, homosexual experience, permissive attitudes toward sexuality, and an external locus of control, while negatively correlating with socialization and control measures (Daitzman and Zuckerman, 1980). Overall previous findings suggest that testosterone and estradiol contribute to impulsive extraversion and expressions of sexuality.

Furthermore, the Disinhibition subscale, associated with impulsive, extraverted, and hedonistic behaviors, is related to both androgens and estrogens (Daitzman and Zuckerman, 1980). Replicated studies on the relationship between androgens, estrogens, and sensation seeking traits, particularly in the Disinhibition subscale of Scale SSS have illustrated that high disinhibitory showed significantly elevated levels of testosterone, 17-β Estradiol, and Estrone compared to low disinhibitory (Daitzman and Zuckerman, 1980). High estradiol levels in males are associated with deviant social attitudes, pathological traits, and deviant/hedonistic behaviors (Daitzman and Zuckerman, 1980). Conversely, low testosterone levels are linked to neurotic introversion and femininity, while high levels are associated with stable, social extraversion (Daitzman and Zuckerman, 1980). Additionally, activity of MAO is highly significant in these studies as they demonstrate that low MAO levels associated with sociability, sensation seeking traits linked to testosterone, and vulnerability to psychiatric disturbances correlated with estradiol in males.

## Conclusion

Overall, our findings help to understand how variations in MAO levels may contribute inclinations to seek novel and thrilling experiences. Future research should explore both genetic and environmental factors that influence MAO levels to better understand the mechanisms involved. The negative association between MAO and SSS may have implications for understanding if a blood platelet biomarker test could have potential for predicting personality characteristics. The correlation between blood platelet MAO levels and sensation seeking, suggests an interesting avenue for understanding and potentially treating maladaptive sensation-seeking behaviors (Sandler et al., 1981; Shih et al., 1999). The data we present here illustrates low correlation on the boredom subscale and high correlation on the experience-seeking subscale. This can be interpreted as individuals who exhibit low levels of sensation seeking in terms of boredom may still seek intense experiences (Sakala et al., 2022). It could also suggest that while they may not actively seek stimulation to avoid boredom, they are more inclined toward novelty, risk-taking, and excitement (Coccini et al., 2002). While the correlation between platelet MAO levels and sensation seeking is intriguing, it is important to note that sensation seeking is just one facet of risky behavior. Other factors such as genetic predisposition, environmental influences, and psychological factors also play significant roles (Shih et al., 1999).

Understanding the relationship between blood platelet MAO levels and sensation seeking offers insights into the underlying mechanisms of maladaptive behaviors. However, there is a need for more studies to explore the discrepancies between *in vivo* and *in vitro* levels and activity of MAO, the mechanism through which gonadal hormones influence behavior via MAO regulation, and to confirm the relationship between platelet and cerebral cortical MAO activities. This is crucial in understanding individual differences in enzyme activity. When coupled with standardized surveys to capture symptoms, behaviors, and other relevant psychopathological data, MAO activity could be used as an integrated approach to correlate activity levels with specific psychiatric conditions. The clinical and research applications of this approach can offer insights leading to novel therapeutic interventions for these disorders.

## Data Availability

The original contributions presented in the study are included in the article/supplementary material, further inquiries can be directed to the corresponding author.
